# Case based approach to the application of standards and Interoperability in practice

**DOI:** 10.1093/eurpub/ckac129.362

**Published:** 2022-10-25

**Authors:** P Hussey, S Das

**Affiliations:** Centre for eIntegrated Care, Dublin City University, Dublin, Ireland

## Abstract

Digital infrastructure and connectivity is layered and requires a number of defined viewpoints in order for safe data flow and use to occur. To address context specific challenges to support communication in health and social care, health informatics standards supporting interoperability are key. In this skills building seminar we will introduce participants to a standards based roadmap for interoperability. We will provide examples of projects and resources detailing the process of engagement to optimise system design for sustainability through a use case based approach.

